# The Pathological Mechanisms of Estrogen-Induced Cholestasis: Current Perspectives

**DOI:** 10.3389/fphar.2021.761255

**Published:** 2021-11-08

**Authors:** Yue Zu, Jinyu Yang, Chengliang Zhang, Dong Liu

**Affiliations:** Tongji Hospital, Tongji Medical College, Huazhong University of Science and Technology, Wuhan, China

**Keywords:** estrogens, bile acid homeostasis, transporter, membrane fluidity, inflammation, intrahepatic cholestasis

## Abstract

Estrogens are steroid hormones with a wide range of biological activities. The excess of estrogens can lead to decreased bile flow, toxic bile acid (BA) accumulation, subsequently causing intrahepatic cholestasis. Estrogen-induced cholestasis (EIC) may have increased incidence during pregnancy, and within women taking oral contraception and postmenopausal hormone replacement therapy, and result in liver injury, preterm birth, meconium-stained amniotic fluid, and intrauterine fetal death in pregnant women. The main pathogenic mechanisms of EIC may include deregulation of BA synthetic or metabolic enzymes, and BA transporters. In addition, impaired cell membrane fluidity, inflammatory responses and change of hepatocyte tight junctions are also involved in the pathogenesis of EIC. In this article, we review the role of estrogens in intrahepatic cholestasis, and outlined the mechanisms of EIC, providing a greater understanding of this disease.

## Introduction

Estrogens are steroid hormones, including several entities, mainly estrone, estriol, 17α-ethinyl estradiol (EE) and the biologically active metabolite 17β-estradiol (E2) (Chen et al., 2019). Estrogens play important roles in cardiovascular system, central nervous system and reproductive system, and participate in the regulation of cholesterol mobilization, electrolyte balance, brain function (Chen et al., 2019). Importantly, estrogens and their metabolites can cause cholestasis in pregnant women and premenopausal women who receive oral contraceptive or use hormone replacement therapy, especially in susceptible people (Schreiber and Simon, 1983; Meier et al., 2008; Pan and Jeong, 2015). In the second or third trimester of sensitive pregnant women, estrogen-induced cholestasis (EIC) is a pregnancy-specific disease with incidence varying between 0.2 and 5.6% and is closely related to the ethnicity and geographic location (Liu and He, 2011; Williamson and Geenes, 2014; Smith and Rood, 2020). In pregnant women, the disease can increase the risk of adverse perinatal outcomes such as preterm birth, meconium-stained amniotic fluid and intrauterine fetal death (Bicocca et al., 2018; Mor et al., 2020). However, there is no specific medicine for clinical treatment of EIC, and the main focus is to protect the liver and reduce cholic acid, so as to improve the clinical pregnancy outcome.

The pathogenesis of EIC is not fully understood. Up to now, bile acid (BA) homeostasis disorder, inflammatory responses, impaired cell membrane fluidity and change of hepatocyte tight junctions are supposed to take part in the development of EIC (Carreras et al., 2007; Mottino et al., 2007; Pan and Jeong, 2015; Xu et al., 2017; El-Hawary et al., 2019; Xiang et al., 2019). Estrogens can induce acute cholestasis by impairing the synthesis, metabolism and transport of bile acids, causing downstream dysfunction of BA homeostasis and a decrease in bile flow (Brouwers et al., 2015; Rezai et al., 2015; Yang et al., 2020). Accordingly, in EIC, accumulated bile acids in the liver can induce oxidative stress and inflammatory reactions, further causing liver injury (Petr et al., 2014). In addition, serum total BAs, total bilirubin, alanine aminotransferase (ALT), alkaline phosphatase (ALP) and aspartate aminotransferase (AST) levels increase in patients with EIC, while total cholesterol levels decrease (Chen et al., 2013; Zhou et al., 2016; Li et al., 2017). In this review, we summarized the main pathological mechanisms of EIC, aiming to provide an important theoretical basis for clinical management of EIC.

## Estrogens

Estrogens are steroid hormones. Three main forms of physiological estrogens, estrone (E1), estradiol (E2, or 17β-estradiol), and estriol (E3) are derived from cholesterol (Conroy et al., 2007; Cui et al., 2013). E2 is the primary reproductive hormone synthesized in the ovary under the stimulation of the follicular stimulating hormone and the luteinizing hormone. E1 and E3 are mostly synthesized in the liver from E2 (Hsu et al., 2017). In terms of estrogenic effect, the most potent and dominant estrogen in humans is E2, with 10-fold more potent than E1 and about 80-fold more potent than E3 (Chung et al., 2010; Gambino et al., 2012; Liu et al., 2018). The structures and properties of these three estrogens are shown in Table 1.

**TABLE 1:** Structures and properties of the estrogens.

Chemical	Structure	Molecular weight	Relative estrogenic activity (yes bioassay)
Estrone (E1)	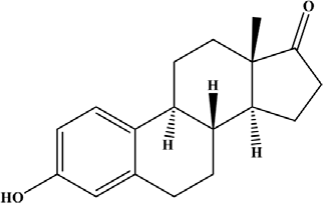	272.39	0.20
Estradiol (E2)	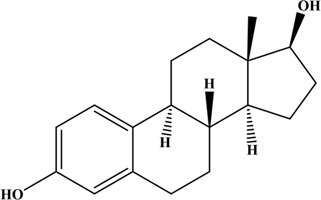	270.37	1.00
Estriol (E3)	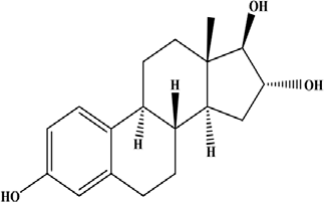	288.39	0.01

Estrogens mediate physiological effects by binding to specific estrogen receptors (ER): estrogen receptor α (ERα), estrogen receptor β (ERβ) and G protein-coupled estrogen receptor 1 (GPER1, also known as GPR30), which are encoded by different genes (Gustafsson, 2003; Nair and Sachdeva, 2018). ERα is predominant receptor. ERβ and GPR30 have also been reported, but there are few studies of them and need to be further explored. Estrogens can interact with intracellular estrogen receptors to exert direct effects by binding to target genes (Freedman and Luisi, 1993). Alternatively, estrogens can activate intracellular signaling cascades via interaction with estrogen receptors (Yasar et al., 2017). Through the above-mentioned ways, estrogens exert a vast range of biological effects in the cardiovascular, musculoskeletal, metabolism, immune, central nervous and reproductive systems (Heldring et al., 2007).

### The Susceptibility of EIC

Our current understanding of EIC during pregnancy is that the elevated levels of estrogens unmask genetic susceptibility in some women, resulting in cholestasis and elevated serum bile acids (Dixon and Williamson, 2016). Clinical studies have found that the susceptibility to cholestasis during pregnancy is associated with heritage, environment and diet (Meier et al., 2008; Pauli-Magnus et al., 2010). The mutations of the hepatobiliary transporter genes, especially bile salt export pump (BSEP), multidrug resistance protein 3 (MDR3) and multidrug-resistance-associated protein 2 (MRP2), can increase the susceptibility to EIC (Sookoian et al., 2008; Anzivino et al., 2013). In addition, the lack of selenium (Se) in the diet may affect the susceptibility to EIC (Kauppila et al., 1987; Reyes et al., 2000). However, the lack of correlation between Se plasma levels and the clinical and biochemical feature of the disease suggests that the role of selenium in the pathogenesis of EIC may be indirect. It is likely that this susceptibility of EIC increases further following the onset of cholestasis.

## Pathophysiological Mechanisms of EIC

### Disturbance of BA Homeostasis

**FIGURE 1:**
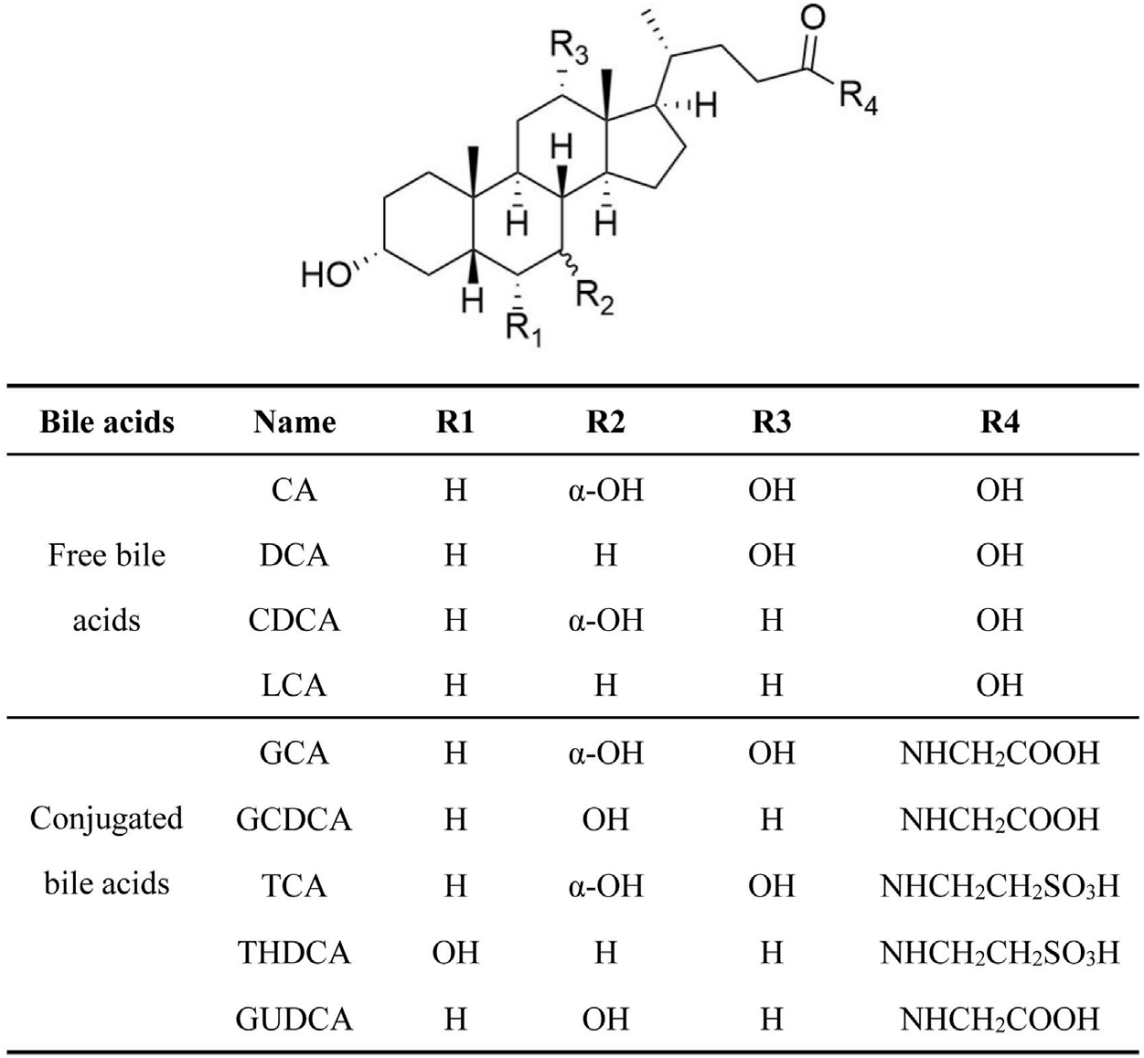
The chemical structure and compositions of BA.

The concentration of BAs in cells and tissues is kept within a certain range under the fine regulation of the normal body functions. This plays an important role in maintaining of the physiological functions of the liver and intestine (Yang et al., 2017; Yang et al., 2019). Bile acids are mainly composed of free and conjugated bile acids (Figure 1), free bile acids: cholic acid (CA), deoxycholic acid (DCA), chenodeoxycholic acid (CDCA) and lithocholic acid (LCA); conjugated bile acids: glycocholic acid (GCA), glycine chenodeoxycholic acid (GCDCA), taurocholic acid (TCA), taurohyodeoxycholic acid (THDCA), and glyuroursodeoxycholic acid (GUDCA), etc.(Carulli et al., 1990). Among them, CA, DCA, CDCA, GCDCA and LCA are hydrophobic bile acids, which can damage hepatocytes. BA homeostasis is tightly related to the process of BA synthesis, metabolism, and transport (Trauner et al., 2017).

Estrogens can reduce the influx and efflux of BA in hepatocytes, resulting in a decrease in bile flow (Yamamoto et al., 2006). Estrogens can also cause abnormal BA compositions, bile obstruction and accumulation of BA, leading to liver toxicity (Yamamoto et al., 2006; Pan and Jeong, 2015; El-Hawary et al., 2019; Dixon and Williamson, 2016). Patients with EIC were associated with a rise in conjugated primary bile acids, particularly the tauroconjugates of CA and CDCA (Table 2) (Tribe et al., 2010). In EIC rats, the hepatic concentration of TCA, DCA and TUDCA was increased, and the level of GCA, GDCA and GUDCA was decreased (Table 2) (Dong et al., 2019; Yang et al., 2020), that resulted in hepatocytes apoptosis and lead to liver damage. Decreases in bile flow and BA homeostasis disturbance by estrogens, have been demonstrated to be related to the disorder of BA enzymes and transporters systems (Henriquez-Hernandez et al., 2007; Meng et al., 2015).

**TABLE 2:** The change of BA compositions in patients and rats with EIC.

Species	BA compositions	References
Human	CA 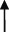 , CDCA 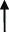	Tribe et al. (2010)
Rat	TCA 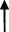 , DCA 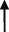 , TUDCA 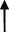	Dong et al. (2019)
Rat	TCA 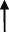 , GCA 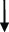 , CA 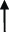 , GUDCA 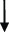 , DCA 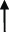 , GDCA 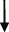 , THDCA 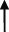	Yang et al. (2020)

#### BA Synthesis and Metabolism in EIC

Estrogens emerge as important regulators of BA synthesis and metabolism through the hepatic feedback mechanisms (Phelps et al., 2019). Bile acids are synthesized from the oxidation of cholesterol in hepatocytes. Cholesterol hydroxylase enzymes play important roles in this process. Above all, three main cholesterol hydroxylase enzymes: cholesterol 7α-hydroxylase (CYP7A1), sterol 12α-hydroxylase (CYP8B1) and sterol 27-hydroxylase (CYP27A1) (Li and Dawson, 2019; Liu and Wang, 2019) are involved in BA synthesis. Studies have found that estrogens can increase the activity CYP7A1, CYP8B1 and CYP27A1, along with small transient increases in BA production (Davis et al., 1986; Chico et al., 1996; Yang et al., 2020). In addition, estrogens can inhibit the expression of BA metabolic enzymes, especially phase II enzymes (such as hydroxysteroid sulfotransferase 2a1, Sult2a1), which in turn decreases the metabolism of bile acids, leading to an increased levels of unconjugated and hydrophobic bile acids in hepatocyte, and a decrease in bile flow (Zamek-Gliszczynski et al., 2006; Zollner and Trauner, 2006; Wang et al., 2019).

#### BA Transporters in EIC

High levels of circulating estrogens are associated with the inhibition of BA transporters in cholestasis (Phelps et al., 2019). The transport of BA depends on hepatic transporters, mainly including ATP-binding cassette (ABC) and solute carrier family (SLC) transporters. ABC transporters can mediate diverse ATP-driven transport processes, mainly including BSEP, MRP2, P-glycoprotein (P-gp/MDR1) ect., (Thoeni et al., 2019). SLC transporters mainly include Na^+^-taurocholate co-transport polypeptide (NTCP), organic anion transporter polypeptides (OATPs) (Beaudoin et al., 2020). Among them, BSEP and MRP2 are the main two BA transporters. Estrogen diminished the transport of BA by down-regulation of these hepatic transporters. Several studies have indicated that a decreased canalicular ATP-dependent BA transport capacity is primarily responsible for the estrogen-induced impairment of BA secretion in the intact liver, resulting in decreased bile flow and increased serum BA and bilirubin (Stieger et al., 2000; Muchova et al., 2015). Estrogens or their metabolites, such as estradiol-17β-d-glucuronic acid (E_2_17G), also impair the expression and function of hepatocyte efflux transporters (BSEP, MRP2, MDR) (Bossard et al., 1993; Stieger et al., 2000; Crocenzi et al., 2001; Yamamoto et al., 2006; Di Guida et al., 2018; Liu et al., 2018). Previous studies have shown that estrogens *trans*-repress BSEP through diminishing peroxisome proliferator-activated receptor-γ (PPARγ) coactivator-1 recruitment (Chen et al., 2015). Moreover, estrogens decrease the expression of multi-drug resistant 2 (MDR2), which causes bile formation disorders (Schreiber and Simon, 1983; Liu et al., 2018). However, Huang et al. found that direct inhibition of BSEP-mediated bile acids transport is not the mechanism for E_2_17G-induced cholestasis, and the process of MRP2-mediated transport is essential for its induction of cholestasis (Huang et al., 2000). Thus, the abnormal expression and function of BA transporters play an important role in the pathogenesis of EIC. However, different estrogens and their metabolites have different effects on the functions of different transporters, and the internal mechanism still needs further study to clarify.

As one of the most important BA sensors in maintaining BA homeostasis, nuclear receptor, farnasol X receptor (FXR) regulates the levels of hepatic transporters to affect BA homeostasis. Estrogens and their metabolites can inhibit the expression of FXR, which decreases the expression of BA transporters in the canalicular membranes of the liver (Wang et al., 2019). This can cause retention of bile acids in hepatocytes and alters the compositions of BA, which subsequently leads to cholestatic liver injury (Lee et al., 2000; Stieger et al., 2000).

**FIGURE 2:**
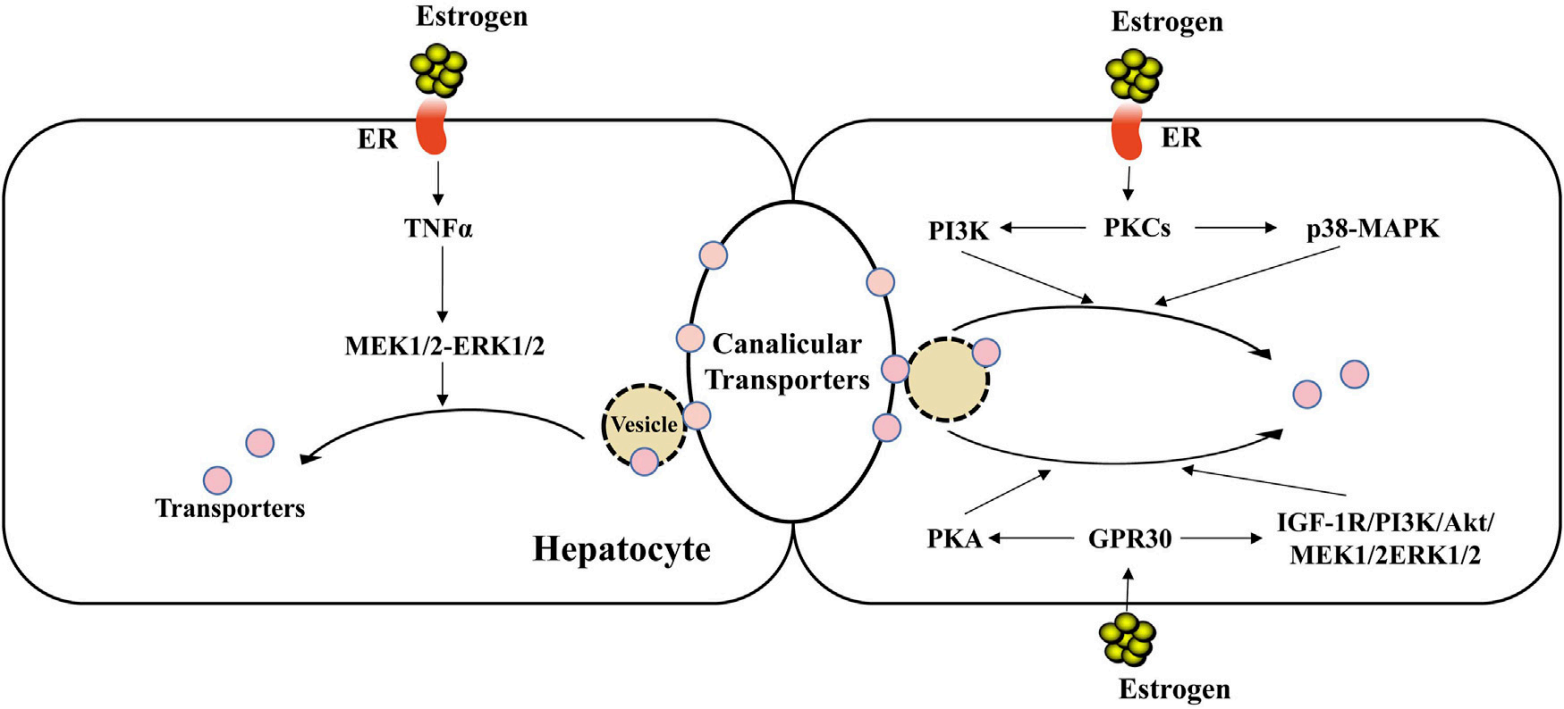
Endocytic internalization of canalicular transporters in EIC. Estrogen can induce the endocytic internalization of canalicular transporters through intracellular signaling cascades, such as PKCs-PI3K/p38 MAPK, GPR30-PKA/TGF1R-PI3K-Akt-MEK1/2-ERK1/2, leading to decreased expression of canalicular transporters and decreased bile flow. Besides, the increased level of hepatic TNFα in EIC may participate in the internalization of transporters by the activation of MEK1/2-ERK1/2 signaling pathway.

In addition, studies have found that cholestasis induced by E_2_17G is related to internalization of the canalicular transporters such as BSEP and MRP2 (Figure 2), which is relevant to bile secretion (Crocenzi et al., 2003; Majer et al., 2007; Miszczuk et al., 2018). Miszczuk, G. S. et al. (Miszczuk et al., 2018) have shown that in E_2_17G-induced cholestasis, the canalicular transporters BSEP and MRP2 undergo exacerbated endocytic internalization caused by a shift of transporters from the caveolin-enriched plasma membrane microdomains (rafts) to the clathrin-enriched ones (non-rafts), resulting in a decrease in the transport activity of them and bile flow.

Estrogens can involve in the endocytosis and internalization of the hepatic transporters through intracellular signaling cascades in EIC. Previous works demonstrated that E_2_17G reduces the expressions of transporters on the membrane through the activation of different signaling proteins to cause their insertion (Mottino et al., 2002; Mottino et al., 2005; Crocenzi et al., 2008). Up to now, there are lots of evidence demonstrated that the protein kinase C (PKC), ERα, p38-MAPK, epidermal growth factor receptor (EGFR) and Src are involved in the endocytosis and internalization of canalicular transporters in EIC (Crocenzi et al., 2008; Boaglio et al., 2012; Andermatten et al., 2019). In the other hand, E_2_17G activates two GPR30-related signal pathway branches: adenylyl cyclase/PKA and insulin-like growth factor receptor-1 (IGF-1R)-phosphoinositide 3 kinase (PI3K)-Akt-MEK1/2-ERK1/2 signaling pathways, which can participate in endocytic internalization of transporters (Crocenzi et al., 2008; Boaglio et al., 2010; Boaglio et al., 2012; Barosso et al., 2016). However, further studies should be required to assess the specific molecular mechanisms mediated by intracellular signaling cascades to impair the localization status of canalicular transporters in EIC.

Besides canalicular transporters, estrogens and their metabolites can also inhibit transporters in the sinusoidal membrane of the liver (Simon et al., 2004; Zhang et al., 2015). Long-term use of estrogens reduced the expression of NTCP and OATPs (Kouzuki et al., 2000). In EIC rats, all basolateral OATPs (1, 2 and 4) were specifically down-regulated on the protein level by 30–40% of the controls, but less pronounced than NTCP (70–80%) (Geier et al., 2003). Therefore, hepatic transporters play an important role in the pathogenesis of EIC.

### Reducing Liver Cell Membrane Fluidity in EIC

The hepatocyte surface membrane plays a pivotal role in BA secretion and excretion (Miccio et al., 1989). EIC has been correlated with structural, biochemical, and physiological abnormalities in the hepatocyte membrane (Miccio et al., 1989; Reyes and Simon, 1993). Estrogens increase the membrane sphingomyelin content and alter the fatty acid composition of the phospholipids, which may be related to the formation of cholestasis (Smith and Gordon, 1988; Malherbe et al., 2020). Studies showed that estrogens can also increase cholesterol content in the cell membrane, resulting in a decrease in membrane fluidity. Also, reduced membrane fluidity may inhibit the movement of transporters on the membrane (Kovanen et al., 1979). Simon et al. found that the decrease in membrane fluidity reduced in bile flow, Na^+^-K^+^-ATPase activity, and the maximum transport rate of BSEP (Simon et al., 1980). In EIC rats, S-adenosylmethionine (SAMe) can increase membrane fluidity and Na^+^-K^+^-ATPase activity, and partially reverse the decrease in bile flow induced by estrogen (Boelsterli et al., 1983). In addition, a defective aquaporin-8 (AQP8) expression in plasma membrane in EIC might be associated with an impairment of the transient osmotic gradients, inducing defective canalicular functional expression of solute transporters together with a reduced canalicular water permeability, and leading to bile secretory dysfunction (Carreras et al., 2007; Lehmann et al., 2008). However, membrane fluidity and Na^+^-K^+^-ATPase activity may be not the only mechanism that involves in the role of hepatocyte membrane in the decreased bile flow (Boelsterli et al., 1983). Bile salts do not cross a lipid membrane. The cholestatic effect may be also produced by an alteration of transporters that are less active in a rigid milieu or there could be an allosteric interaction between cholesterol and BSEP, which should be verified. Therefore, the relationship between membrane fluidity and bile flow has yet to be fully determined.

### Oxidative Stress and Inflammatory Responses in EIC

Excess estrogens can induce oxidative stress (Diaz et al., 2019) and pro-inflammatory cytokine expression in liver (Martin-Millan and Castaneda, 2013). Study has shown that estrogens significantly decrease the content of steroid cyanide, peroxide and glutathione (GSH) in liver cells, leading to the membrane lipid peroxidation and free radicals production, which in turn enhances the oxidative damage in EIC (Yu et al., 2016). Indeed, EIC rats show a massive oxidative stress and lipid peroxidation as evidenced by a significant drop in the hepatic GSH content and a subsequent increase in the hepatic thiobarbituric acid reactive substances (TBARS) levels (Chen et al., 2019). Besides, ROS may also cause several effects that could be implied in cholestasis, including canalicular transporter disinsertion (Ciriaci et al., 2019).

Rujuan Dai et al. (Dai et al., 2009) have revealed that *in vivo* massive estrogen exposure promotes inflammatory responses that include enhanced secretion of Th1 related cytokines (IFNγ, IL-12, IL-1β), inflammatory chemokines (MCP-1 and MCP-5), and induction of inducible nitric oxide synthase (iNOS) and cyclooxygenases-2 (Cox-2), leading to the liver damage. Estrogens lead to an obvious increase in hepatic tumor necrosis factor (TNF)-α and hepatic myeloperoxidase (MPO) levels in the liver tissues by 346 and 232%, respectively (Wadie et al., 2021). The increased level of hepatic TNFα can participate in the internalization of MRP2 by the activation of NADPH oxidase, ROS and MEK1/2-ERK1/2 signaling pathways (Ciriaci et al., 2019). Study has confirmed that the MEK-ERK signaling pathway involved in the endocytosis of MRP2 in EIC (Boaglio et al., 2012). This implies that TNFα/ROS/MEK-ERK pathway may also participate in the endocytosis of transporters in EIC (Dai et al., 2009). Besides, Bach1/Nrf2 pathway and NF-κB pathway may be also involved in liver inflammatory damage during EIC (Petrone et al., 2014; Muchova et al., 2015). Therefore, estrogens can induce oxidative stress and inflammation, promoting the progress of EIC.

### Disrupted Hepatocyte Tight Junctions in EIC

Hepatocyte tight junctions (TJs) are composed of multiple proteins that are anchored directly or indirectly to the actin-based cytoskeleton (Chen et al., 2009). The integrity of TJs is of utmost importance for holding back diffusion of bile components from the canalicular to the blood. Estrogens can affect hepatocyte polarity and, in addition, disrupt TJs (Mottino et al., 2007; Chen et al., 2009). Estrogens can cause the deterioration of TJs, which can cause disturbances in the osmotic gradient from bile to plasma and lead to the failure of the apical-basolateral diffusion barrier (Mottino et al., 2007; Zollner and Trauner, 2008).

## Conclusion and Perspectives

**FIGURE 3:**
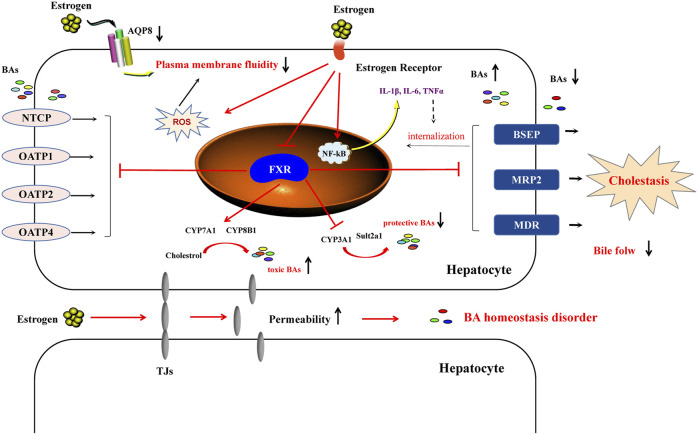
The main pathological mechanism of estrogen-induced cholestasis. In the liver, estrogen enters the plasma membrane of the hepatocyte to interact with estrogen receptors (ERα and ERβ), which can inhibit the expression of FXR and then regulate the expression of downstream genes. Estrogens increase the activity of bile acid synthase (CYP7A1 and CYP8B1) and inhibit their metabolic enzymes (CYP3A1, CYP3A11 and Sult2a1), as well as promote the increase of bile acid content in hepatocytes. Regarding transporters, estrogens reduce the levels of BSEP and MRP2, as well as inhibit NTCP and OATPs, resulting in a decrease in bile flow. In addition, estrogen-induced cholestasis is related to the impaired membrane fluidity. The increase of ROS and inflammatory response can also exacerbate estrogen-induced cholestasis liver injury. Estrogen can increase the permeability of TJs, leading to the imbalance of BA homeostasis and promoting the progress of EIC.

In conclusion, EIC is a complex pathological process. In-depth knowledge of the main pathological mechanisms is crucial to ensure clear understanding of EIC. Currently, the main pathological mechanisms include BA homeostasis dysfunction, poor liver cell membrane fluidity, oxidative stress, inflammatory responses, and change of hepatocyte tight junctions (Figure 3). These factors contribute to the accumulation of BAs in livers, which in turn results in cholestasis.

Despite the increasing attention to EIC, its pathological mechanism still need to be further explored. For instance, the influences of estrogens on the location and function of BA transporters during cholestasis have not been fully elucidated, which is critical for the development of potential therapeutic agents. Due to the complexity and change of EIC’s etiology and complications, the response of existing treatment drugs (such as ursodeoxycholic acid) is not sensitive. Therefore, an in-depth understanding of the mechanisms of EIC will help to develop new drug targets.

## References

[B1] Andermatten R. B. Ciriaci N. Schuck V. S. Di Siervi N. Razori M. V. Miszczuk G. S. (2019). Sphingosine 1-phosphate Receptor 2/adenylyl Cyclase/protein Kinase A Pathway Is Involved in Taurolithocholate-Induced Internalization of Abcc2 in Rats. Arch. Toxicol. 93 (8), 2279–2294. 10.1007/s00204-019-02514-6 31300867

[B2] Anzivino C. Odoardi M. R. Meschiari E. Baldelli E. Facchinetti F. Neri I. (2013). ABCB4 and ABCB11 Mutations in Intrahepatic Cholestasis of Pregnancy in an Italian Population. Dig. Liver Dis. 45 (3), 226–232. 10.1016/j.dld.2012.08.011 23022423

[B3] Barosso I. R. Zucchetti A. E. Miszczuk G. S. Boaglio A. C. Taborda D. R. Roma M. G. (2016). EGFR Participates Downstream of ERα in Estradiol-17β-D-Glucuronide-Induced Impairment of Abcc2 Function in Isolated Rat Hepatocyte Couplets. Arch. Toxicol. 90 (4), 891–903. 10.1007/s00204-015-1507-8 25813982

[B4] Beaudoin J. J. Bezençon J. Sjöstedt N. Fallon J. K. Brouwer K. L. R. (2020). Role of Organic Solute Transporter Alpha/Beta in Hepatotoxic Bile Acid Transport and Drug Interactions. Toxicol. Sci. 176 (1), 34–35. 10.1093/toxsci/kfaa052 32294204 PMC7357176

[B5] Bicocca M. J. Sperling J. D. Chauhan S. P. (2018). Intrahepatic Cholestasis of Pregnancy: Review of Six National and Regional Guidelines. Eur. J. Obstet. Gynecol. Reprod. Biol. 231, 180–187. 10.1016/j.ejogrb.2018.10.041 30396107

[B6] Boaglio A. C. Zucchetti A. E. Sánchez Pozzi E. J. Pellegrino J. M. Ochoa J. E. Mottino A. D. (2010). Phosphoinositide 3-kinase/protein Kinase B Signaling Pathway Is Involved in Estradiol 17β-D-Glucuronide-Induced Cholestasis: Complementarity with Classical Protein Kinase C. Hepatology 52 (4), 1465–1476. 10.1002/hep.23846 20815017

[B7] Boaglio A. C. Zucchetti A. E. Toledo F. D. Barosso I. R. Sánchez Pozzi E. J. Crocenzi F. A. (2012). ERK1/2 and P38 MAPKs Are Complementarily Involved in Estradiol 17ß-D-Glucuronide-Induced Cholestasis: Crosstalk with cPKC and PI3K. PLoS One 7 (11), e49255. 10.1371/journal.pone.0049255 23166621 PMC3498151

[B8] Boelsterli U. A. Rakhit G. Balazs T. (1983). Modulation by S-Adenosyl-L-Methionine of Hepatic Na+,K+-ATPase, Membrane Fluidity, and Bile Flow in Rats with Ethinyl Estradiol-Induced Cholestasis. Hepatology 3 (1), 12–17. 10.1002/hep.1840030102 6295906

[B9] Bossard R. Stieger B. O'Neill B. Fricker G. Meier P. J. (1993). Ethinylestradiol Treatment Induces Multiple Canalicular Membrane Transport Alterations in Rat Liver. J. Clin. Invest. 91 (6), 2714–2720. 10.1172/JCI116511 8514879 PMC443336

[B10] Brouwers L. Koster M. P. Page-Christiaens G. C. Kemperman H. Boon J. Evers I. M. (2015). Intrahepatic Cholestasis of Pregnancy: Maternal and Fetal Outcomes Associated with Elevated Bile Acid Levels. Am. J. Obstet. Gynecol. 212 (1), 100. 10.1016/j.ajog.2014.07.026 25046809

[B11] Carreras F. I. Lehmann G. L. Ferri D. Tioni M. F. Calamita G. Marinelli R. A. (2007). Defective Hepatocyte Aquaporin-8 Expression and Reduced Canalicular Membrane Water Permeability in Estrogen-Induced Cholestasis. Am. J. Physiol. Gastrointest. Liver Physiol. 292 (3), G905–G912. 10.1152/ajpgi.00386.2006 17110522

[B12] Carulli N. Loria P. Bertolotti M. Carubbi F. Tripodi A. Abate N. (1990). Effects of Bile Acid Pool Composition on Hepatic Metabolism of Cholesterol in Man. Ital. J. Gastroenterol. 22 (2), 88–96. 2131939

[B13] Chen C. Gong X. Yang X. Shang X. Du Q. Liao Q. (2019). The Roles of Estrogen and Estrogen Receptors in Gastrointestinal Disease. Oncol. Lett. 18 (6), 5673–5680. 10.3892/ol.2019.10983 31788039 PMC6865762

[B14] Chen J. Zhao K. N. Liu G. B. (2013). Estrogen-induced Cholestasis: Pathogenesis and Therapeuticimplications. Hepatogastroenterology 60 (126), 1289–1296. 10.5754/hge121061 23933920

[B15] Chen W. Gao X. X. Ma L. Liu Z. B. Li L. Wang H. (2019). Obeticholic Acid Protects against Gestational Cholestasis-Induced Fetal Intrauterine Growth Restriction in Mice. Oxid. Med. Cel. Longev. 2019, 7419249. 10.1155/2019/7419249 PMC6885290 31827696

[B16] Chen X. Zhang C. Wang H. Xu J. Duan Z. H. Zhang Y. (2009). Altered Integrity and Decreased Expression of Hepatocyte Tight Junctions in Rifampicin-Induced Cholestasis in Mice. Toxicol. Appl. Pharmacol. 240 (1), 26–36. 10.1016/j.taap.2009.06.022 19577586

[B17] Chen Y. Vasilenko A. Song X. Valanejad L. Verma R. You S. (2015). Estrogen and Estrogen Receptor-α-Mediated Transrepression of Bile Salt Export Pump. Mol. Endocrinol. 29 (4), 613–626. 10.1210/me.2015-1014 25675114 PMC4399276

[B18] Chico Y. Fresnedo O. Botham K. Lacort M. Ochoa B. (1996). Regulation of Bile Acid Synthesis by Estradiol and Progesterone in Primary Cultures of Rat Hepatocytes. Exp. Clin. Endocrinol. Diabetes 104 (2), 137–144. 10.1055/s-0029-1211435 8740937

[B19] Chung S. H. Franceschi S. Lambert P. F. (2010). Estrogen and ERalpha: Culprits in Cervical Cancer?. Trends Endocrinol. Metab. 21 (8), 504–511. 10.1016/j.tem.2010.03.005 20456973 PMC2914219

[B20] Ciriaci N. Andermatten R. B. Razori M. V. Schuck V. S. Miszczuk G. S. Medeot A. C. (2019). Role of ERK1/2 in TNFα-Induced Internalization of Abcc2 in Rat Hepatocyte Couplets. Biochem. Pharmacol. 164, 311–320. 10.1016/j.bcp.2019.04.024 31026445

[B21] Conroy O. Sáez A. E. Quanrud D. Ela W. Arnold R. G. (2007). Changes in Estrogen/anti-Estrogen Activities in Ponded Secondary Effluent. Sci. Total Environ. 382 (2-3), 311–323. 10.1016/j.scitotenv.2007.04.033 17543371

[B22] Crocenzi F. A. Mottino A. D. Cao J. Veggi L. M. Pozzi E. J. Vore M. (2003). Estradiol-17beta-D-glucuronide Induces Endocytic Internalization of Bsep in Rats. Am. J. Physiol. Gastrointest. Liver Physiol. 285 (2), G449–G459. 10.1152/ajpgi.00508.2002 12702498

[B23] Crocenzi F. A. Sánchez Pozzi E. J. Pellegrino J. M. Favre C. O. Rodríguez Garay E. A. Mottino A. D. (2001). Beneficial Effects of Silymarin on Estrogen-Induced Cholestasis in the Rat: a Study *In Vivo* and in Isolated Hepatocyte Couplets. Hepatology 34 (2), 329–339. 10.1053/jhep.2001.26520 11481618

[B24] Crocenzi F. A. Sánchez Pozzi E. J. Ruiz M. L. Zucchetti A. E. Roma M. G. Mottino A. D. (2008). Ca(2+)-dependent Protein Kinase C Isoforms Are Critical to Estradiol 17beta-D-Glucuronide-Induced Cholestasis in the Rat. Hepatology 48 (6), 1885–1895. 10.1002/hep.22532 18972403 PMC3004396

[B25] Cui J. Shen Y. Li R. (2013). Estrogen Synthesis and Signaling Pathways during Aging: from Periphery to Brain. Trends Mol. Med. 19 (3), 197–209. 10.1016/j.molmed.2012.12.007 23348042 PMC3595330

[B26] Dai R. Phillips R. A. Karpuzoglu E. Khan D. Ahmed S. A. (2009). Estrogen Regulates Transcription Factors STAT-1 and NF-kappaB to Promote Inducible Nitric Oxide Synthase and Inflammatory Responses. J. Immunol. 183 (11), 6998–7005. 10.4049/jimmunol.0901737 19890039 PMC2782783

[B27] Davis R. A. Elliott T. S. Lattier G. R. Showalter R. B. Kern F. (1986). Regulation of Bile Acid Synthesis via Direct Effects on the Microsomal Membrane. Biochemistry 25 (7), 1632–1636. 10.1021/bi00355a028 3707898

[B28] Di Guida F. Pirozzi C. Magliocca S. Santoro A. Lama A. Russo R. (2018). Galactosylated Pro-drug of Ursodeoxycholic Acid: Design, Synthesis, Characterization, and Pharmacological Effects in a Rat Model of Estrogen-Induced Cholestasis. Mol. Pharm. 15 (1), 21–30. 10.1021/acs.molpharmaceut.7b00626 29140706

[B29] Díaz A. López-Grueso R. Gambini J. Monleón D. Mas-Bargues C. Abdelaziz K. M. (2019). Sex Differences in Age-Associated Type 2 Diabetes in Rats-Role of Estrogens and Oxidative Stress. Oxid. Med. Cel. Longev. 2019, 6734836. 10.1155/2019/6734836 PMC6476064 31089412

[B30] Dixon P. H. Williamson C. (2016). The Pathophysiology of Intrahepatic Cholestasis of Pregnancy. Clin. Res. Hepatol. Gastroenterol. 40 (2), 141–153. 10.1016/j.clinre.2015.12.008 26823041

[B31] Dong R. Wang J. Gao X. Wang C. Liu K. Wu J. (2019). Yangonin Protects against Estrogen-Induced Cholestasis in a Farnesoid X Receptor-dependent Manner. Eur. J. Pharmacol. 857, 172461. 10.1016/j.ejphar.2019.172461 31220436

[B32] El-Hawary S. S. Ali Z. Y. Younis I. Y. (2019). Hepatoprotective Potential of Standardized Ficus Species in Intrahepatic Cholestasis Rat Model: Involvement of Nuclear Factor-Κb, and Farnesoid X Receptor Signaling Pathways. J. Ethnopharmacol. 231, 262–274. 10.1016/j.jep.2018.11.026 30458280

[B33] Freedman L. P. Luisi B. F. (1993). On the Mechanism of DNA Binding by Nuclear Hormone Receptors: a Structural and Functional Perspective. J. Cel. Biochem. 51 (2), 140–150. 10.1002/jcb.240510205 8440748

[B34] Gambino Y. P. Pérez Pérez A. Dueñas J. L. Calvo J. C. Sánchez-Margalet V. Varone C. L. (2012). Regulation of Leptin Expression by 17beta-Estradiol in Human Placental Cells Involves Membrane Associated Estrogen Receptor Alpha. Biochim. Biophys. Acta 1823 (4), 900–910. 10.1016/j.bbamcr.2012.01.015 22310000

[B35] Geier A. Dietrich C. G. Gerloff T. Haendly J. Kullak-Ublick G. A. Stieger B. (2003). Regulation of Basolateral Organic Anion Transporters in Ethinylestradiol-Induced Cholestasis in the Rat. Biochim. Biophys. Acta 1609 (1), 87–94. 10.1016/s0005-2736(02)00657-0 12507762

[B36] Gustafsson J. A. (2003). What Pharmacologists Can Learn from Recent Advances in Estrogen Signalling. Trends Pharmacol. Sci. 24 (9), 479–485. 10.1016/S0165-6147(03)00229-3 12967773

[B37] Heldring N. Pike A. Andersson S. Matthews J. Cheng G. Hartman J. (2007). Estrogen Receptors: How Do They Signal and what Are Their Targets. Physiol. Rev. 87 (3), 905–931. 10.1152/physrev.00026.2006 17615392

[B38] Henríquez-Hernández L. A. Flores-Morales A. Santana-Farré R. Axelson M. Nilsson P. Norstedt G. (2007). Role of Pituitary Hormones on 17alpha-Ethinylestradiol-Induced Cholestasis in Rat. J. Pharmacol. Exp. Ther. 320 (2), 695–705. 10.1124/jpet.106.113209 17108234

[B39] Hsu L. H. Chu N. M. Kao S. H. (2017). Estrogen, Estrogen Receptor and Lung Cancer. Int. J. Mol. Sci. 18 (8). 10.3390/ijms18081713 PMC5578103 28783064

[B40] Huang L. Smit J. W. Meijer D. K. Vore M. (2000). Mrp2 Is Essential for Estradiol-17beta(beta-D-Glucuronide)-Induced Cholestasis in Rats. Hepatology 32 (1), 66–72. 10.1053/jhep.2000.8263 10869290

[B41] Kauppila A. Korpela H. Mäkilä U. M. Yrjänheikki E. (1987). Low Serum Selenium Concentration and Glutathione Peroxidase Activity in Intrahepatic Cholestasis of Pregnancy. Br. Med. J. (Clinical Res. Edition) 294 (6575), 150–152. 10.1136/bmj.294.6565.150 PMC1245162 3109544

[B42] Kouzuki H. Suzuki H. Stieger B. Meier P. J. Sugiyama Y. (2000). Characterization of the Transport Properties of Organic Anion Transporting Polypeptide 1 (Oatp1) and Na(+)/taurocholate Cotransporting Polypeptide (Ntcp): Comparative Studies on the Inhibitory Effect of Their Possible Substrates in Hepatocytes and cDNA-Transfected COS-7 Cells. J. Pharmacol. Exp. Ther. 292 (2), 505–511. 10640286

[B43] Kovanen P. T. Brown M. S. Goldstein J. L. (1979). Increased Binding of Low Density Lipoprotein to Liver Membranes from Rats Treated with 17 Alpha-Ethinyl Estradiol. J. Biol. Chem. 254 (22), 11367–11373. 10.1016/s0021-9258(19)86495-5 227868

[B44] Lee J. M. Trauner M. Soroka C. J. Stieger B. Meier P. J. Boyer J. L. (2000). Expression of the Bile Salt export Pump Is Maintained after Chronic Cholestasis in the Rat. Gastroenterology 118 (1), 163–172. 10.1016/s0016-5085(00)70425-2 10611165

[B45] Lehmann G. L. Larocca M. C. Soria L. R. Marinelli R. A. (2008). Aquaporins: Their Role in Cholestatic Liver Disease. World J. Gastroenterol. 14 (46), 7059–7067. 10.3748/wjg.14.7059 19084912 PMC2776835

[B46] Li J. Dawson P. A. (2019). Animal Models to Study Bile Acid Metabolism. Biochim. Biophys. Acta Mol. Basis Dis. 1865 (5), 895–911. 10.1016/j.bbadis.2018.05.011 29782919 PMC6242766

[B47] Li X. Liu R. Luo L. Yu L. Chen X. Sun L. (2017). Role of AMP-Activated Protein Kinase α1 in 17α-Ethinylestradiol-Induced Cholestasis in Rats. Arch. Toxicol. 91 (1), 481–494. 10.1007/s00204-016-1697-8 27090119 PMC5069111

[B48] Liu J. Hou L. L. Zhao C. Y. (2018). Effect of YHHJ on the Expression of the Hepatocellular Bile Acid Transporters Multidrug Resistance-Associated Protein 2 and Bile Salt export Pump in Ethinylestradiol-Induced Cholestasis. Exp. Ther. Med. 15 (4), 3699–3704. 10.3892/etm.2018.5891 29563980 PMC5858118

[B49] Liu X. Wang Y. (2019). [An Overview of Bile Acid Synthesis and its Physiological and Pathological Functions]. Yi Chuan 41 (5), 365–374. 10.16288/j.yczz.19-011 31106772

[B50] Liu X. Xue R. Yang C. Gu J. Chen S. Zhang S. (2018). Cholestasis-induced Bile Acid Elevates Estrogen Level via Farnesoid X Receptor-Mediated Suppression of the Estrogen Sulfotransferase SULT1E1. J. Biol. Chem. 293 (33), 12759–12769. 10.1074/jbc.RA118.001789 29929982 PMC6102144

[B51] Liu X. H. He J. (2011). [Pay More Attention to Standardizing the Diagnosis and Treatment of Intrahepatic Cholestasis of Pregnancy]. Zhonghua Fu Chan Ke Za Zhi 46 (5), 321–323. 21733364

[B52] Majer F. Trnka L. Vítek L. Jirkovská M. Marecek Z. Smíd F. (2007). Estrogen-induced Cholestasis Results in a Dramatic Increase of B-Series Gangliosides in the Rat Liver. Biomed. Chromatogr. 21 (5), 446–450. 10.1002/bmc.743 17357127

[B53] Malherbe J. A. J. Garas G. Khor T. S. Macquillan G. C. (2020). Delayed Fulminant Hepatic Failure from Dydrogesterone-Related *In Vitro* Fertilization Therapy Requiring Liver Transplantation during Pregnancy. Am. J. Case Rep. 21, e925690. 10.12659/AJCR.925690 32938902 PMC7520868

[B54] Martín-Millán M. Castañeda S. (2013). Estrogens, Osteoarthritis and Inflammation. Jt. Bone Spine 80 (4), 368–373. 10.1016/j.jbspin.2012.11.008 23352515

[B55] Meier Y. Zodan T. Lang C. Zimmermann R. Kullak-Ublick G. A. Meier P. J. (2008). Increased Susceptibility for Intrahepatic Cholestasis of Pregnancy and Contraceptive-Induced Cholestasis in Carriers of the 1331T>C Polymorphism in the Bile Salt export Pump. World J. Gastroenterol. 14 (01), 38–45. 10.3748/wjg.14.38 18176959 PMC2673389

[B56] Meng Q. Chen X. Wang C. Liu Q. Sun H. Sun P. (2015). Protective Effects of Alisol B 23-Acetate via Farnesoid X Receptor-Mediated Regulation of Transporters and Enzymes in Estrogen-Induced Cholestatic Liver Injury in Mice. Pharm. Res. 32 (11), 3688–3698. 10.1007/s11095-015-1727-x 26040663

[B57] Miccio M. Orzes N. Lunazzi G. C. Gazzin B. Corsi R. Tiribelli C. (1989). Reversal of Ethinylestradiol-Induced Cholestasis by Epomediol in Rat. The Role of Liver Plasma-Membrane Fluidity. Biochem. Pharmacol. 38 (20), 3559–3563. 10.1016/0006-2952(89)90128-7 2554925

[B58] Miszczuk G. S. Barosso I. R. Larocca M. C. Marrone J. Marinelli R. A. Boaglio A. C. (2018). Mechanisms of Canalicular Transporter Endocytosis in the Cholestatic Rat Liver. Biochim. Biophys. Acta Mol. Basis Dis. 1864 (4), 1072–1085. 10.1016/j.bbadis.2018.01.015 29355600

[B59] Mor M. Shmueli A. Krispin E. Bardin R. Sneh-Arbib O. Braun M. (2020). Intrahepatic Cholestasis of Pregnancy as a Risk Factor for Preeclampsia. Arch. Gynecol. Obstet. 301 (3), 655–664. 10.1007/s00404-020-05456-y 32034507

[B60] Mottino A. D. Cao J. Veggi L. M. Crocenzi F. Roma M. G. Vore M. (2002). Altered Localization and Activity of Canalicular Mrp2 in Estradiol-17beta-D-Glucuronide-Induced Cholestasis. Hepatology 35 (6), 1409–1419. 10.1053/jhep.2002.33327 12029626

[B61] Mottino A. D. Crocenzi F. A. Pozzi E. J. Veggi L. M. Roma M. G. Vore M. (2005). Role of Microtubules in Estradiol-17beta-D-Glucuronide-Induced Alteration of Canalicular Mrp2 Localization and Activity. Am. J. Physiol. Gastrointest. Liver Physiol. 288 (2), G327–G336. 10.1152/ajpgi.00227.2004 15374814

[B62] Mottino A. D. Hoffman T. Crocenzi F. A. Sánchez Pozzi E. J. Roma M. G. Vore M. (2007). Disruption of Function and Localization of Tight Junctional Structures and Mrp2 in Sustained Estradiol-17beta-D-Glucuronide-Induced Cholestasis. Am. J. Physiol. Gastrointest. Liver Physiol. 293 (1), G391–G402. 10.1152/ajpgi.00496.2006 17463180

[B63] Muchova L. Vanova K. Suk J. Micuda S. Dolezelova E. Fuksa L. (2015). Protective Effect of Heme Oxygenase Induction in Ethinylestradiol-Induced Cholestasis. J. Cel. Mol. Med. 19 (5), 924–933. 10.1111/jcmm.12401 PMC4420596 25683492

[B64] Nair S. Sachdeva G. (2018). Estrogen Matters in Metastasis. Steroids 138, 108–116. 10.1016/j.steroids.2018.07.006 30031855

[B65] Pan X. Jeong H. (2015). Estrogen-Induced Cholestasis Leads to Repressed CYP2D6 Expression in CYP2D6-Humanized Mice. Mol. Pharmacol. 88 (1), 106–112. 10.1124/mol.115.098822 25943116 PMC4468640

[B66] Pauli-Magnus C. Meier P. J. Stieger B. (2010). Genetic Determinants of Drug-Induced Cholestasis and Intrahepatic Cholestasis of Pregnancy. Semin. Liver Dis. 30 (2), 147–159. 10.1055/s-0030-1253224 20422497

[B67] Petr T. Smíd V. Kučerová V. Váňová K. Leníček M. Vítek L. (2014). The Effect of Heme Oxygenase on Ganglioside Redistribution within Hepatocytes in Experimental Estrogen-Induced Cholestasis. Physiol. Res. 63 (3), 359–367. 10.33549/physiolres.932665 24564601

[B68] Petrone A. B. Simpkins J. W. Barr T. L. (2014). 17β-estradiol and Inflammation: Implications for Ischemic Stroke. Aging Dis. 5 (5), 340–345. 10.14336/AD.2014.0500340 25276492 PMC4173799

[B69] Phelps T. Snyder E. Rodriguez E. Child H. Harvey P. (2019). The Influence of Biological Sex and Sex Hormones on Bile Acid Synthesis and Cholesterol Homeostasis. Biol. Sex. Differ. 10 (1), 52. 10.1186/s13293-019-0265-3 31775872 PMC6880483

[B70] Reyes H. Báez M. E. González M. C. Hernández I. Palma J. Ribalta J. (2000). Selenium, Zinc and Copper Plasma Levels in Intrahepatic Cholestasis of Pregnancy, in normal Pregnancies and in Healthy Individuals, in Chile. J. Hepatol. 32 (4), 542–549. 10.1016/s0168-8278(00)80214-7 10782901

[B71] Reyes H. Simon F. R. (1993). Intrahepatic Cholestasis of Pregnancy: an Estrogen-Related Disease. Semin. Liver Dis. 13 (3), 289–301. 10.1055/s-2007-1007357 8235718

[B72] Rezai S. Lam J. Henderson C. E. (2015). Intrahepatic Cholestasis of Pregnancy: Maternal and Fetal Outcomes Associated with Elevated Bile Acid Levels. Am. J. Obstet. Gynecol. 213 (1), 114. 10.1016/j.ajog.2015.03.040 25818669

[B73] Schreiber A. J. Simon F. R. (1983). Estrogen-induced Cholestasis: Clues to Pathogenesis and Treatment. Hepatology 3 (4), 607–613. 10.1002/hep.1840030422 6305818

[B74] Simon F. R. Fortune J. Iwahashi M. Qadri I. Sutherland E. (2004). Multihormonal Regulation of Hepatic Sinusoidal Ntcp Gene Expression. Am. J. Physiol. Gastrointest. Liver Physiol. 287 (4), G782–G794. 10.1152/ajpgi.00379.2003 15361361

[B75] Simon F. R. Gonzalez M. Sutherland E. Accatino L. Davis R. A. (1980). Reversal of Ethinyl Estradiol-Induced Bile Secretory Failure with Triton WR-1339. J. Clin. Invest. 65 (4), 851–860. 10.1172/JCI109737 6244335 PMC434472

[B76] Smith D. D. Rood K. M. (2020). Intrahepatic Cholestasis of Pregnancy. Clin. Obstet. Gynecol. 63 (1), 134–151. 10.1097/GRF.0000000000000495 31764000

[B77] Smith D. J. Gordon E. R. (1988). Role of Liver Plasma Membrane Fluidity in the Pathogenesis of Estrogen-Induced Cholestasis. J. Lab. Clin. Med. 112 (6), 679–685. 3193023

[B78] Sookoian S. Castaño G. Burgueño A. Gianotti T. F. Pirola C. J. (2008). Association of the Multidrug-Resistance-Associated Protein Gene (ABCC2) Variants with Intrahepatic Cholestasis of Pregnancy. J. Hepatol. 48 (1), 125–132. 10.1016/j.jhep.2007.08.015 17997497

[B79] Stieger B. Fattinger K. Madon J. Kullak-Ublick G. A. Meier P. J. (2000). Drug- and Estrogen-Induced Cholestasis through Inhibition of the Hepatocellular Bile Salt export Pump (Bsep) of Rat Liver. Gastroenterology 118 (2), 422–430. 10.1016/s0016-5085(00)70224-1 10648470

[B80] Thoeni C. Waldherr R. Scheuerer J. Schmitteckert S. Roeth R. Niesler B. (20192019). Expression Analysis of ATP-Binding Cassette Transporters ABCB11 and ABCB4 in Primary Sclerosing Cholangitis and Variety of Pediatric and Adult Cholestatic and Noncholestatic Liver Diseases. Can. J. Gastroenterol. Hepatol. 2019, 1085717. 10.1155/2019/1085717 PMC6925824 31886153

[B81] Trauner M. Fuchs C. D. Halilbasic E. Paumgartner G. (2017). New Therapeutic Concepts in Bile Acid Transport and Signaling for Management of Cholestasis. Hepatology 65 (4), 1393–1404. 10.1002/hep.28991 27997980

[B82] Tribe R. M. Dann A. T. Kenyon A. P. Seed P. Shennan A. H. Mallet A. (2010). Longitudinal Profiles of 15 Serum Bile Acids in Patients with Intrahepatic Cholestasis of Pregnancy. Am. J. Gastroenterol. 105 (3), 585–595. 10.1038/ajg.2009.633 19904249

[B83] Wadie W. Mohamed A. H. Masoud M. A. Rizk H. A. Sayed H. M. (2021). Protective Impact of Lycopene on Ethinylestradiol-Induced Cholestasis in Rats. Naunyn Schmiedebergs Arch. Pharmacol. 394 (3), 447–455. 10.1007/s00210-020-01980-5 33034714

[B84] Wang J. Fu T. Dong R. Wang C. Liu K. Sun H. (2019). Hepatoprotection of Auraptene from the Peels of Citrus Fruits against 17α-Ethinylestradiol-Induced Cholestasis in Mice by Activating Farnesoid X Receptor. Food Funct. 10 (7), 3839–3850. 10.1039/c9fo00318e 31210195

[B85] Williamson C. Geenes V. (2014). Intrahepatic Cholestasis of Pregnancy. Obstet. Gynecol. 124 (1), 120–133. 10.1097/AOG.0000000000000346 24901263

[B86] Xiang D. Yang J. Liu Y. He W. Zhang S. Li X. (2019). Calculus Bovis Sativus Improves Bile Acid Homeostasis via Farnesoid X Receptor-Mediated Signaling in Rats with Estrogen-Induced Cholestasis. Front. Pharmacol. 10, 48. 10.3389/fphar.2019.00048 30774596 PMC6367682

[B87] Xu Y. J. Yu Z. Q. Zhang C. L. Li X. P. Feng C. Y. Lei K. (2017). Protective Effects of Ginsenosides on 17[Formula: See Text]-Ethynyelstradiol-Induced Intrahepatic Cholestasis via Anti-oxidative and Anti-inflammatory Mechanisms in Rats. Am. J. Chin. Med. 45 (8), 1613–1629. 10.1142/S0192415X17500872 29121800

[B88] Yamamoto Y. Moore R. Hess H. A. Guo G. L. Gonzalez F. J. Korach K. S. (2006). Estrogen Receptor Alpha Mediates 17alpha-Ethynylestradiol Causing Hepatotoxicity. J. Biol. Chem. 281 (24), 16625–16631. 10.1074/jbc.M602723200 16606610

[B89] Yang J. Xiang D. Xiang D. He W. Liu Y. Lan L. (2020). Baicalin Protects against 17α-Ethinylestradiol-Induced Cholestasis via the Sirtuin 1/Hepatic Nuclear Receptor-1α/Farnesoid X Receptor Pathway. Front. Pharmacol. 10, 1685. 10.3389/fphar.2019.01685 32116682 PMC7026019

[B90] Yang T. Khan G. J. Wu Z. Wang X. Zhang L. Jiang Z. (2019). Bile Acid Homeostasis Paradigm and its Connotation with Cholestatic Liver Diseases. Drug Discov. Today 24 (1), 112–128. 10.1016/j.drudis.2018.09.007 30244079

[B91] Yang T. Mei H. Xu D. Zhou W. Zhu X. Sun L. (2017). Early Indications of ANIT-Induced Cholestatic Liver Injury: Alteration of Hepatocyte Polarization and Bile Acid Homeostasis. Food Chem. Toxicol. 110, 1–12. 10.1016/j.fct.2017.09.051 28986171

[B92] Yaşar P. Ayaz G. User S. D. Güpür G. Muyan M. (2017). Molecular Mechanism of Estrogen-Estrogen Receptor Signaling. Reprod. Med. Biol. 16 (1), 4–20. 10.1002/rmb2.12006 29259445 PMC5715874

[B93] Yu L. Liu X. Li X. Yuan Z. Yang H. Zhang L. (2016). Protective Effects of SRT1720 via the HNF1α/FXR Signalling Pathway and Anti-inflammatory Mechanisms in Mice with Estrogen-Induced Cholestatic Liver Injury. Toxicol. Lett. 264, 1–11. 10.1016/j.toxlet.2016.10.016 27818225

[B94] Zamek-Gliszczynski M. J. Hoffmaster K. A. Nezasa K. Tallman M. N. Brouwer K. L. (2006). Integration of Hepatic Drug Transporters and Phase II Metabolizing Enzymes: Mechanisms of Hepatic Excretion of Sulfate, Glucuronide, and Glutathione Metabolites. Eur. J. Pharm. Sci. 27 (5), 447–486. 10.1016/j.ejps.2005.12.007 16472997

[B95] Zhang G. Zhou Y. Rao Z. Qin H. Wei Y. Ren J. (2015). Effect of Yin-Zhi-Huang on Up-Regulation of Oatp2, Ntcp, and Mrp2 Proteins in Estrogen-Induced Rat Cholestasis. Pharm. Biol. 53 (3), 319–325. 10.3109/13880209.2014.918156 25420584

[B96] Zhou F. Gao B. Deng C. Huang G. Xu T. Wang X. (2016). Dynamic Expression of Corticotropin-Releasing Hormone and Urocortin in Estrogen Induced-Cholestasis Pregnant Rat. Reprod. Toxicol. 65, 179–186. 10.1016/j.reprotox.2016.07.019 27492720

[B97] Zollner G. Trauner M. (2008). Mechanisms of Cholestasis. Clin. Liver Dis. 12 (1), 1–vii. 10.1016/j.cld.2007.11.010 18242495

[B98] Zollner G. Trauner M. (2006). Molecular Mechanisms of Cholestasis. Wien Med. Wochenschr 156 (13-14), 380–385. 10.1007/s10354-006-0312-7 16937039

[B99] Zucchetti A. E. Barosso I. R. Boaglio A. Pellegrino J. M. Ochoa E. J. Roma M. G. (2011). Prevention of Estradiol 17beta-D-Glucuronide-Induced Canalicular Transporter Internalization by Hormonal Modulation of cAMP in Rat Hepatocytes. Mol. Biol. Cel. 22 (20), 3902–3915. 10.1091/mbc.E11-01-0047 PMC3192868 21865596

